# Enhancing Yield and Physiological Performance by Foliar Applications of Chemically Inert Mineral Particles in a Rainfed Vineyard under Mediterranean Conditions

**DOI:** 10.3390/plants12071444

**Published:** 2023-03-24

**Authors:** Despoina G. Petoumenou

**Affiliations:** Laboratory of Viticulture, Department of Agriculture, Crop Production and Rural Environment, University of Thessaly, Fytokou Street, 38446 Volos, Greece; petoumenou@uth.gr

**Keywords:** climate change, kaolin, zeolite, temperature, leaf gas exchange parameters, water use efficiency, total phenols, *Vitis vinifera* L.

## Abstract

One of the biggest environmental challenges that most of the traditional and modern grape-growing areas are facing is the frequency, severity, and unpredictability of extreme weather events as a result of climate change. Sustainable tools such as chemically inert mineral particles could be a valid alternative for the promotion of environmentally-friendly viticultural techniques to enhance yield, improve physiological processes, and increase tolerance to biotic/abiotic stressors and grape quality. In regard to this concept, the effects of kaolin (KL) and zeolite (ZL) application was tested in the rosé grapevine cultivar Roditis, field-and rainfed, under the Mediterranean conditions of central Greece. In a two-year trial, the whole vine canopy was sprayed with kaolin and zeolite until runoff at a dose of 3% (*w*/*v*) twice throughout the growing season; the first at the beginning of veraison and the second one week later; treatment of the untreated control plants was also performed (C). The assimilation rate in morning and midday, the stomatal conductance, and the WUEi of the leaves of the treated and untreated plants were monitored one day after each application and at harvest. During the same time period of the day (i.e., morning and midday) in July, August, and September, the leaf temperature near the fruit zone was also recorded. At harvest, the yield parameters, cluster characteristics, grape composition, and incidence (%) of sunburned and dehydrated berries as well as berries infected by *Plasmopara viticola* and *Lobesia botrana* were recorded. The results showed that KL and ZL application decreased leaf temperature during the growing season until harvest compared to the control treatment, which resulted in an improvement in physiological parameters such as net photosynthesis and intrinsic water use efficiency. At harvest, the KL- and ZL-treated vines showed increased yield due to an increasing cluster and berry fresh weight. On the other hand, the KL and ZL application did not affect the sugar concentration and pH of the must and increased the total acidity and decreased the total phenolic compound content, but only in the first year of the experiments. Furthermore, the incidence of sunburn necrosis, dehydrated berries, and infected berries was significantly lower in the treated vines compared to the control vines. These results confirm the promising potential of kaolin and zeolite applications as a stress mitigation strategy during the summer period, with the ability to protect grapevine plants, enhance yield, and maintain or improve fruit quality in rainfed Mediterranean vineyards.

## 1. Introduction

Grapevine (*Vitis vinifera* L.) is the third most economically important fruit crop worldwide [1], playing a decisive socioeconomic and cultural role, but it is unfortunately under threat from climate change in the coming decades [2,3].

The major part of grapevine growing areas is characterized by a Mediterranean climate with warm and dry summers. In these particular areas, the grapevine cultivars have increasingly been exposed to intense and extreme climatic events (e.g., heatwaves and prolonged drought) during recent years, with undesirable effects on yield and berry quality [4,5,6,7,8,9,10,11]. These phenomena are expected to increase in intensity and frequency [12] and are considered of utmost importance in non-irrigated or rainfed vineyards. In the rainfed Mediterranean wine regions, water stress can be particularly severe during summer, especially as this period is followed by the winter and spring dry periods. This issue has so far been addressed with the irrigation of vineyards, a process which has expanded in traditional Mediterranean dry regions such as Spain, France, Portugal, and Italy [13]. This cropping system of vineyards is expected to increase for a variety of reasons including climate change and the reconsideration of irrigation restrictions in many traditionally rainfed regions.

The predicted increase in the periodicity of extreme weather events (e.g., heatwaves and prolonged droughts), along with the simultaneous incidence of high luminosity and extreme temperatures during the summer, may impact grapevine photosynthetic capacity [14,15,16] and fruit quality [15,16,17,18,19].

The Roditis (*Vitis vinifera* L.) is the second most widely cultivated Greek autochthonous grapevine variety after the Savvatiano, with almost 10,000 hectares [20]. It is included in many of the protected appellations such as the Patras, Anchialos, Plagies Melitona, and the traditional wine appellation Retsina, which is the most famous traditional Greek wine. One of the rising problems in the cultivation of this variety is the yield loss and the altered wine identity during the hottest vintages. In fact, the combination of rising temperatures and decreasing precipitation in relation to the grapevine phenological stage can cause total yield loss [4,21,22]. Moreover, even in the case where yield is not affected, these conditions can lead to unbalanced grape maturity with significant increase in total soluble solids in the must and/or low acidity levels, and consequently to modified characteristics of the wine style of a given region [23]. The resulting wines are characterized by more alcohol content and less acidity, aroma, and color, consequently failing to meet consumers’ preferences [3,13]. Indeed, the high content of alcohol in the wines is no longer in accordance with consumers’ demand and the World Health Organization’s action plan, which guides a global strategy to reduce the unhealthy use of alcohol worldwide [24].

Under these circumstances, grapevine growers across the world are obliged more than ever in the past to identify agronomic strategies to maintain and improve the competitiveness and sustainability of vineyards subjected to these stressors. There is a large number of potential tools for protecting grapevines from these adverse conditions, including the utilization of more tolerant rootstocks and cultivars, changes in canopy management, the promotion of adequate irrigation strategies, and the application of special protective compounds such as kaolin (KL) and zeolite (ZL).

Reflective and chemically inert mineral particles are characterized by their ability to reflect infrared, PAR, and ultraviolet radiation, rendering them a potentially viable agronomic tool in commercial vineyards [2,25]. In fact, it is well known that, due to modifications in the fruit and leaf tissues after spraying, KL may exercise a repulsive effect against arthropods in different crops [26,27,28,29,30,31,32,33,34]. Moreover, KL is also an efficient tool in reducing fruit and leaf sunburn damage in several fruit crop species, such as apple [35,36], pomegranate [37,38], and mango [39] as well as grapevine [40]. In recent years, there has been an increasing number of studies, especially dealing with olive and grapevine, which investigate plant physiological performance after KL applications as a means to alleviate multiple stresses during the summer period. Therefore, it has been suggested that treating grapevine leaves with KL may result in higher photochemical efficiency of the PSII system [41] or a higher sucrose transport and phloem loading capacity [42]. A positive response was also registered in olive plants, where leaves sprayed with KL recorded less oxidative damage [43,44]. Consequently, the cooling and protective effect of KL on fruits could be a strategy to maintain and/or improve pre-harvest and post-harvest fruit quality [45,46].

Zeolites are crystalline aluminosilicates of alkali and alkaline earth elements composed of a tetrahedral framework of SiO_4_ and AlO_4_. Due to their potentially promising characteristics (e.g., nontoxicity, high cation exchange capacity, eco-friendliness, cost-effectiveness, and abundant availability), zeolites have been considered as preeminent sorbent materials for the agro-food industry [47,48,49]. Several earlier findings reported the effects of zeolite application on soil properties—especially nutrient and water retention capacity, crop yield, and heavy metal toxicity [50]—as well as zeolites’ role in plant physiology parameters, crop yield, and crop quality [51,52].

Although white and rosé grapevine varieties are more susceptible to climate change than red ones due to their low heat demands [53], to the best of the author’s knowledge there is a lack of studies that explore simultaneously the KL and ZL effects on these varieties.

For this reason, the aim of the present study was to assess the effects of KL and ZL applications on the rosé grapevine variety Roditis in terms of plant physiology and berry quality. For this purpose, single-leaf gas exchange parameters were evaluated one day after each application and at harvest; the leaf temperature was also recorded. Finally, at the harvesting stage, pest and disease incidence, yield, and fruit quality characteristics were estimated.

## 2. Results

### 2.1. Environmental Conditions and Leaf Temperature

The analysis of the main meteorological parameters indicates that both the 2018 and the 2019 vintages were warmer than the average of the last 60 years (Table 1). In detail, April, May, and July 2018 were hotter than the average of the last 60 years for the region (+3, +2.2, +0.9 °C, respectively). For the rest of the months, the values were within the range of the last 60 years. On the other hand, June, July, August, and October 2019 were warmer than usual (+2.1, +0.8, +1.8 and 1.2 °C, respectively).

In 2018, rains occurred mostly in May (+34 mm than the 60-year average) and June (+137 mm), whereas April was drier than usual (−31.1 mm compared to the 60-year average). In 2019, rains were higher than usual and occurred in June, August, and September (+86 mm, +91 mm and −48 mm than the 60-year average, respectively). Overall, both the 2018 and 2019 growing seasons (from 1 April to 31 October) were wetter than the 60 previous years (+112 mm and +226 mm, respectively), and a non-uniform distribution of precipitations was recorded during the summer period in both years (Table 1).

**Figure 1:**
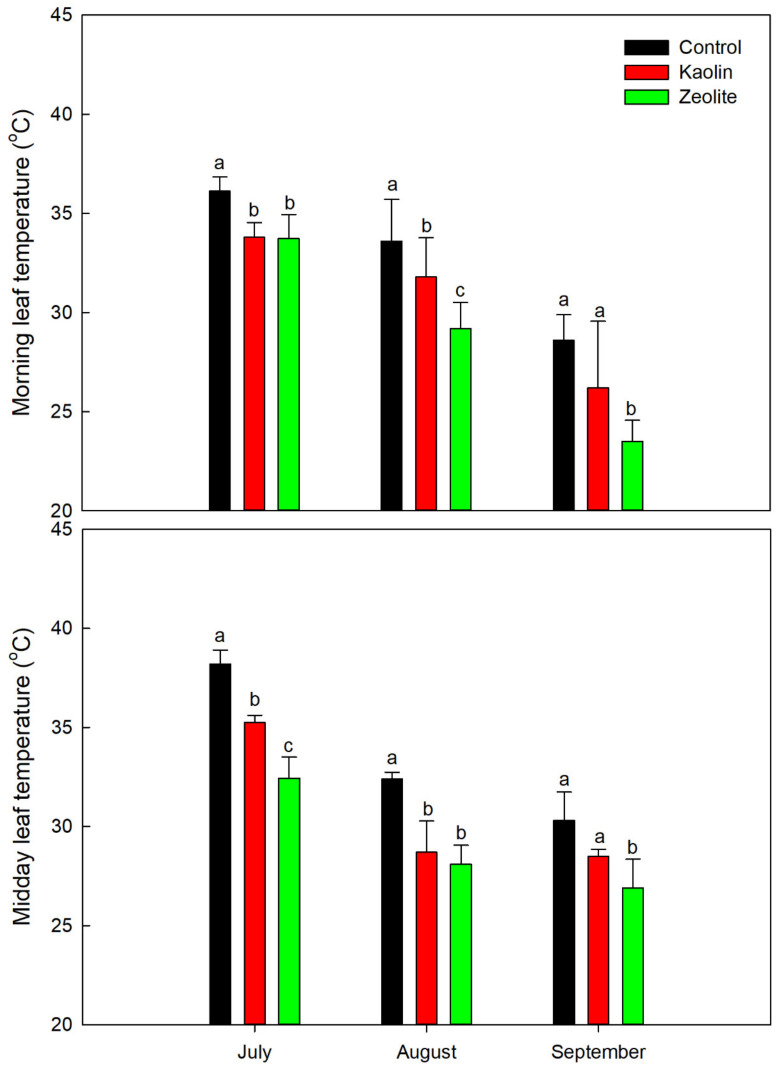
Single leaf temperatures of the control-, kaolin-, and zeolite-treated vines measured in the morning and at midday in July (one day after treatment, 193 days of the year (DOY), BBCH-79-81, berry touch complete—beginning of ripening), August (DOY 213, BBCH-83, berries brightening in color), and September (270 DOY, BBC-89, at harvest) in 2018. Columns are means and vertical bars represent standard deviation of measurements on ten fully expanded leaves per treatment. Mean values of the same month with different lowercase letters (a, b, c) represent significant differences between treatments (*p* ≤ 0.05, *t*-test).

Chemical inert particle films (kaolin and zeolite) affected several grapevine physiological parameters. As presented in Figure 1, the leaf temperature, which can be considered as the first indicator of plant health status under warm weather conditions, was positively affected by KL and ZL applications. The control leaf temperatures reached peaks of 38.2 °C at midday in July, and the control plants generally showed higher leaf temperatures in the morning and midday periods and in all stages than the treated ones (KL or ZL). On the other hand, the KL- and ZL-treated leaves showed a decrease of −2.3 °C in leaf temperature in the morning in July (just one day after application). Moreover, the same applications (KL and ZL) showed a decrease of −1.8 °C and −4.4 °C and −2.4 °C and −5.1 °C in leaf temperature in the morning in the veraison and harvest stages, respectively. More pronounced differences were registered at the same stages at midday. Noticeably, the leaf temperature of the zeolite-treated vines showed a significantly lower temperature at midday in July (−5.8 °C vs. −2.9 °C of kaolin-treated leaves), at veraison (−4.3 °C vs. −3.7 °C of the kaolin-treated leaves), and at harvest (−3.4 °C vs. −1.8 °C of the kaolin-treated leaves) as compared to the untreated control vines. Therefore, it could be suggested that only ZL-treated vines resulted in significantly lower leaf temperatures until August compared to both the control and KL-treated vines.

### 2.2. Leaf Gas Exchange Parameters and Vine Water Status

Regarding the leaf gas exchange parameters (Table 2) of zeolite treated vines, the results showed high values of net photosynthesis (*P*_n_), stomatal conductance (*g*_s_), and intrinsic water use efficiency (WUEi) in all the sampling stages and in both periods of the day (morning and midday) compared to the control and kaolin-treated vines. The ZL treatment led to significantly higher *P*_N_ and WUEi values in all stages and periods in 2018.

As regards the ψ_stem_, the results of kaolin and zeolite showed differences compared to the control vines only in the morning period in July and September, whereas in the midday in July only the ZL-treated plants showed lower values of ψ_stem_ compared to the C- and KL-treated plants. No significant differences in midday stem water potential at harvest were found (Table 2).

### 2.3. Yield Components and Fruit Composition at Harvest

The KL and ZL applications increased the yield per vine in both vintages compared to the C vines, while the same chemically inert particles positively affected the berry weight (Table 3). Precisely and for the 2018 growing period, the ZL and KL vines presented 28.3% and 24.6% higher yield compared to the control treatment, respectively. The same trend was registered in 2019, where the ZL and KL vines presented 21.6% and 9.3% higher yield compared to the C vines, respectively. No differences in the number of clusters per vine and the number of berries per cluster were found between the treatments. Grape sugar accumulation and must pH were not influenced by the KL and ZL applications in both years. However, the ZL and KL grapes presented 12.2% and 8.2% higher total acidity in both the 2018 and 2019 growing periods, respectively. Phenolic compound content was affected by the KL and ZL applications only in 2018 (Table 3).

### 2.4. Grape Health Status at Harvest

**Figure 2:**
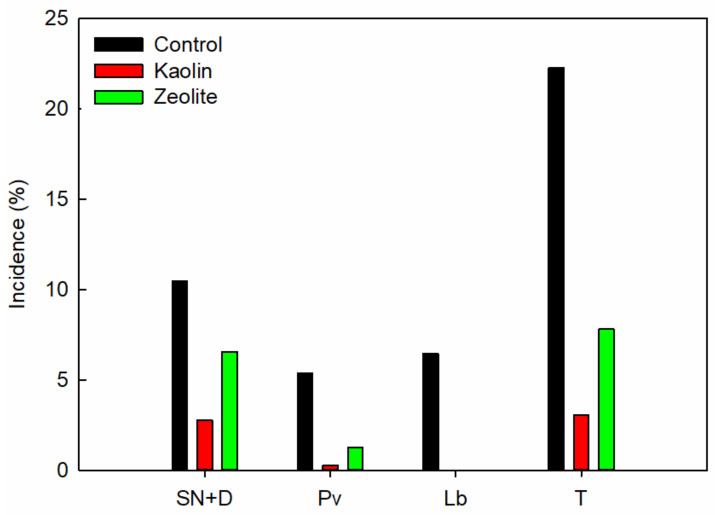
Incidence (%) of sunburn necrosis and dehydration (SN+D), *Plasmopara viticola* (PV) and *Lobesia botrana* (Lb) pests of control, kaolin, and zeolite vines at harvest 2018. T: the total amount of clusters affected by both abiotic and biotic factors.

The control vines recorded 10% of clusters presenting sunburn necrosis and dehydrated berries, whereas the KL- and ZL-treated vines showed the values of 3% and 7%, respectively (Figure 2). The control clusters affected by *Plasmopara viticola* had an average of 5% infected berries, while only 0.3% and 1% of the KL and ZL vines were affected, respectively. The results also revealed that the KL and ZL applications inhibited the incidence of *Lobesia botrana* pests, whereas 6.5% of the clusters were affected in the case of the control vines. Therefore, our data suggest that the application of KL and ZL markedly reduced the incidence of abiotic and biotic factors by 87% and 65%, respectively, compared to the control vines.

## 3. Discussion

A profitable and high-standard vineyard cropping is deeply dependent on climate conditions and weather fluctuations and is particularly reliant on management practices [3].

In rainfed viticultural systems and under the global warming scenario, canopy management is crucial in order to reduce water losses. This interaction between the agroecosystem and grapevines creates unique combinations that characterize the grape quality and the final wine style. In fact, the grower’s choices during the growing season are crucial in allowing them to cope with adverse circumstances and ultimately to maintain continuous productivity and profitability. Thus, the main goal of vineyard management should be the maintenance an equalized microclimate that further promotes optimum canopy growth and vine physiology and ensures high-quality products.

The present study evaluated the importance of alternative agronomic techniques such as zeolite and kaolin foliar applications as a potential climate adaptation tool that may guarantee the production and quality of berry cv. Roditis vines in a rainfed Mediterranean vineyard.

This variety presents at least two different clones which differ in berry color. The wines produced by this particular cultivar are characterized by high acidity and low polyphenolic accumulation—characteristics which can be compromised under different terroirs and limiting conditions (water scarcity and/or high summer temperatures). It is therefore necessary to adopt new strategies in order to guarantee satisfying grape production without compromising fruit and wine quality.

Based on previous studies, kaolin particle film material is highly reflective to ultraviolet wavelengths [54,55]; therefore, the reduction in leaf temperature in KL- and ZL-treated vines is to be expected. Our single-leaf thermal readings confirm the KL and ZL leaf cooling capacity in increasingly limited vine water status (Figure 1, Table 2). A similar scenario was reported by Shellie and King [56] and by Frioni et al. [57]. In contrast, Valentini et al. [58] reported that in a rainfed vineyard of the Sangiovese cultivar, KL and ZL spraying reduced the berry temperature but not the leaf temperature of vines at the second year of the experiment, probably due to rootstock x cultivar interaction and sufficient soil water content; this, however, did not affect the overall plant physiology status.

In C and KL leaves, a nonstomatal limitation to photosynthesis processes was evident, as the Pn and WUEi decreased (Table 2), whereas the literature suggests contrary data for KL application on leaf gas exchange parameters. These differences could be due to different ambient and/or leaf tissue conditions, since when the environment exercises some limiting factor for the plant, KL treatment might result in a positive effect on leaf photosynthesis [41,59,60,61]. When kaolin is applied in rainy and low-irradiance environments, photosynthesis is reduced [43,62]. In general, a loss of KL effectiveness in terms of stomatal conductance and net photosynthetic rate has been registered under severe stress conditions in both grapevines [5,63] and olive trees [64]. Considering the unique features of each species and cultivar, the effectiveness of KL application against stress effects in terms of leaf gas exchange parameters is higher under moderately stressful than under extreme conditions. Nevertheless, in some cases this phenomenon was not reflected in the whole-canopy photosynthesis [65,66], probably due to the ability of KL to alter the light distribution within the canopy in relation to the canopy architecture [66,67,68].

In our study kaolin always decreased photosynthesis, independently to plant water status. Conversely, zeolite maintained a high photosynthetic performance throughout the growing season, confirming the findings of Shellie and King [56] and De Smedt et al. [69] in apple trees. Indeed, the ZL particles protected the leaves from high temperatures and led to a better WUEi, which is highly linked to a lower abscisic acid accumulation, and consequently is able to reduce possible damages by heat stress and sunburn injury.

The present results revealed that kaolin and zeolite, applied twice at 3% (*w*/*v*) concentration at the beginning of berry touch, differ regarding the persistence of mineral coating on leaves. Compared to kaolin, zeolite showed a higher photosynthetic performance on leaves which lasted until harvest. These data are confirmed for the first time in grapevines and show that zeolite effects on vine physiology are not correlated to plant water status (Table 2). Conversely, kaolin highly reduced canopy carbon assimilation as compared to zeolite, and the values for the same parameters were similar to the control vines. This finding could be explained by the higher leaf-to-air temperature observed for the KL when compared to the ZL treatment. It was also confirmed that ZL application is able to increase WUE_i_ more than KL when the drought stress becomes severe (Table 2). A higher WUE can be achieved through lower stomatal conductance and transpiration, caused by stomatal closure, by higher photosynthetic capacity or a combination of both [70,71,72]. This probably explains the findings of this study where both treatments increased yield. The higher yield per plant could be also attributable to the decrease in damage to the cell membranes by lipid peroxidation [60,73], as well as by the delay in leaf senescence of the grapevines sprayed with KL and ZL. However, contradictory results regarding yield in response to KL application can be found in the literature [56,64,67,74,75] due to the interaction of environmental factors with the species and cultivars and/or the plant canopy architecture.

Regarding berry composition, grape sugar concentration was similar for all the treatments, whereas titratable acidity was significantly affected by kaolin and zeolite applications (Table 3), contrary to a previous report [57,59]. Although concentrations of individual organic acids such as malic and tartaric acid were not registered, it can be hypothesized that the positive effect of coating material applications on must acidity could be related to lower malic acid respiration rates.

Results in previous studies about the effect of kaolin on berry quality are contradictory [42,56,75,76,77,78,79], probably due to different climatic conditions (i.e., arid and high temperate or humid areas); by contrast, studies on zeolite are scarce.

In this study, kaolin and zeolite sprays reduced berry total phenolic compound content only in the first year of the experiment (Table 3). As reported by Dinis et al. [80], spraying 5% kaolin on grape at the onset of ripening constrained the hydroxyl radicals and boosted antioxidant compounds such as phenolics, favonoids, anthocyanins, vitamin C, and all key metabolites in the berries relative to the control. This contradiction might be attributable to the lower concentration (3%; *w*/*v*) of the coating film used in our experiment or because our vines were relieved from water stress after the cooling effect of the particle films as compared to the control, since polyphenol content in grapes can increase under water stress conditions [81,82]. Moreover, particularly high yield levels, such as registered in the KL and ZL vines (Table 3), might be detrimental to fruit quality and especially to total phenolic compounds and anthocyanins contents [83]. However, phenolic compounds are also responsible for phenomena such as the darkening of white wines, oxidation, and bitterness, thus exerting a negative effect on wine quality. Therefore, the results observed in this study indicate that kaolin and zeolite particle films must be applied according to winery standards due to their impact on the oenological performance of grapes and in relation to the grapevine cultivar.

The kaolin particle film was initially performed for reducing diseases and attacks from arthropod pests due to its repellent effect [28,29,30,31,32,33,34,84]. Our results confirm that KL and ZL may reduce the incidence of diseases, sunburn necrosis, and dehydration of grapes, confirming their repellent benefits as reported in other crops, e.g., pomegranate, apple, pear, and olive trees [29,85,86,87]. Due to their white color (after application, Supplementary Figure S1) and light reflection, kaolin and zeolite can reduce the attractiveness of plant tissues and fruits to pests [65]. Moreover, these coating films on fruit adhere to insects’ feet, thus confounding their movement, feeding, and egg laying. Indeed, the use of kaolin and zeolite at a concentration of 3% (*w*/*v*) eliminated the *Lobesia botrana* infestation on grapes in this study (Figure 2).

## 4. Materials and Methods

### 4.1. Weather Data

The weather parameters were recorded by the Hellenic Air Force automated weather station located nearby the experimental vineyard; monthly average air temperature and precipitation from 1 April to 31 October in 2018, in 2019, and during the last sixty years (1957–2017) were considered.

### 4.2. Plant Material and Treatment Layout

This trial was carried out in the 2018 and 2019 growing seasons on a six-year-old commercial vineyard, cv. Roditis (*Vitis vinifera* L.), grafted on Richter 110 rootstock. The vineyard was located in the characteristically flat land of the protected designation of origin (PDO) zone in Nea Anchialos (Thessaly), Greece. The soil was classified as sandy-loam, and the vines were spaced at 1.15 m within the row and at 2.60 m between rows and trained to a bilateral Royat system with approximately eight to ten spurs per vine retained at winter pruning. The vines were rainfed, and fertilization and cultural practices (berry thinning, leaf removal, shoot thinning, and shoot trimming) were conducted according to local practices. Each year, all the wines were uniformed both for number of shoots and for clusters at the flowering stage, and a standard disease control program was applied in order to control powdery mildew, downy mildew, and botrytis bunch rot.

The experimental design consisted of 75 vines organized in a randomized complete block design (RCBD), with three blocks composed of 25 vines each and selected from three different rows. The three selected rows were separated by one untreated row in order to limit drift effects. At each block, the following three independent treatments were applied: (i) control vines (C) treated only with water, (ii) kaolin (KL)-sprayed vines (Surround^®^ WP, 95% kaolin, 5% inert ingredients, AgNova Technologies Pty Ltd., Australia), and (iii) zeolite (ZL)-sprayed vines. The zeolite was natural, 0.09–0.02 mm in size, and originated from Bulgaria. The purified material contained 80% clinoptilolite [(Na,K,Ca)_2-3_Al_3_(Al,Si)_2_Si_13_O_36_·12(H_2_O)] with the following composition (in weight %): SiO_2_ 67.29, Al_2_O_3_ 12.51, CaO 2.94, MgO 0.62, K_2_O 3.30, Fe_2_O_3_ 1.42, MnO 0.026, P_2_O_5_ < 0.05 (no data for the presence of toxic elements are available).

Both formulations of kaolin and zeolite were mixed and diluted in water at 3% (*w*/*v*) concentration and were sprayed twice each year. The first applications took place on 11 July (DOY [day of year] 192, BBCH 79-81, according to Lorenz et al. [88]) and 13 July (DOY 194) for the years 2018 and 2019, respectively. After the applications, a strong rainfall took place, so it was decided to repeat the treatments, and a second application was performed seven days later, on 16 July (DOY 199) and 20 July (DOY 121) for the two years of the trial, respectively.

All the suspensions were carefully applied with a battery-powered backpack sprayer on both canopy sides for a full canopy spray until the runoff was recorded.

### 4.3. Gas Exchange and Leaf Temperature Measurements

The first year of the experiment, leaf net photosynthesis (Pn) and stomatal conductance (g_s_) of well-exposed and mature primary leaves were measured one day after the treatments, namely on 12 July (DOY 193, BBCH 79-81) and at harvest, 13 September (DOY 256, BBCH 89), using a LC Pro+ portable photosynthesis system (ADC Bioscientific Ltd., Hoddesdon, UK). Readings were performed on sunny clear days in the morning (09:00–10:00 am) and at midday (13:00–14:00 pm) under constant saturating light (≅1600 μmol m^−2^⋅s^−1^) on seven fully sun-exposed leaves per treatment and at the middle portion (sixth node) of the main shoot. Concurrently, on the same leaves, the intrinsic water use efficiency (WUE_i_) was calculated as the Pn/g_s_ ratio, and the leaf temperature (Tleaf) was recorded using an infrared laser thermometer point temperature gun (Type K, MASTECH MS6541, China). The stem water potential was measured with a pressure chamber (Soil Moisture Equipment Corp., Santa Barbara, CA, USA) as described by Scholander et al. [89].

### 4.4. Yield Components, Grape Composition, and Pest and Disease Incidence

Harvest was performed on 13 September (DOY 256) in 2018 and 2 October (DOY 275) in 2019 when the sugar accumulation on ND vines reached 20 degrees Brix (°Bx). All the experimental vines were individually hand-picked, and the total number of clusters per vine was recorded; at the same time, the yield per vine was recorded with a portable field scale (KERN & Sohn GmbH, CH15K20, Balingen, Germany). Then stereo microscopic observations (stereo microscope Stemi DRC, G/436124, Zeiss, Germany) were applied to all the clusters per treatment, and the percentage of berries attacked by *Lobesia botrana* was recorded. In 2018 and at the same harvested clusters, *Botrytis cinerea* and *Plasmopara viticola* symptoms were detected too. The incidence was expressed as the percentage (%) of affected berries.

Thereafter, ten representative clusters per vine were immediately weighed, and their berries were separated from the rachis and counted; the berry weight was recorded and the average berry weight calculated. Fifty berries per cluster were collected for further analyses, and the remaining berries were crushed and the must obtained in order to determine the following berry quality characteristics. The concentration of total soluble solids (TSS) was determined using a digital handheld “pocket” refractometer PAL (Atago Co., Ltd., Tokyo, Japan) and expressed in °Brix at 20 °C. A digital HI-2002 Edge pH meter (Hanna Instruments, Rhode Island, USA) was used to measure the must pH, and the values were expressed in pH units. The titratable acidity (TA) was determined by titration of the grape juice with a 0.1 N sodium hydroxide (NaOH) solution in the presence of a bromothymol blue indicator and expressed as g/L of tartaric acid equivalents. The remaining berries were frozen at −20 °C, and after a few days, the total skin phenolic compound content was determined as described by Slinkard and Singleton [90], and their concentration was expressed as milligrams per kilo of fresh berry weight (mg/Kg).

### 4.5. Statistical Analysis

The data were subjected to one-way ANOVA using the Sigmaplot package, v.12 (SystatSoftware Inc., San Jose, CA, USA). The significance of the differences between the mean values of each treatment was determined according to the *t*-test at *p* ≤ 0.05 and *p* ≤ 0.01. The figures were illustrated with the SigmaPlot package.

## 5. Conclusions

Preserving yield and grape quality within a climate change scenario is a subject of growing interest in many viticultural areas worldwide.

The objective of this study was to evaluate the effects of kaolin and zeolite particle film applications on the vine physiology, yield, and grape quality of one of the most important rosé grapevine cultivars in Greece, cv. Roditis.

The beneficial effects of zeolite foliar application were more pronounced than kaolin as regarding leaf temperature, single leaf photosynthesis, stomatal conductance, and WUE_i_ throughout the growing season. On both vintages, yield and total acidity were also higher in the kaolin- and zeolite-treated fruits than in the control fruits. Moreover, both particle films confirmed their validity against the most common grapevine diseases and pests. Early-medium (i.e., around the beginning of ripening) applications of kaolin and zeolite coating films can be a useful and sustainable tool to maintain and/or increase yield in rainfed vineyards. Nevertheless, further research is needed to elucidate mechanisms of action involved and metabolite dynamics after the zeolite and kaolin applications and the interaction of these minerals with other agronomic practices in order to improve their effectiveness under extreme stress conditions.

## Figures and Tables

**Table 1:** Monthly average temperature (T; °C) and rainfall (mm) recorded for the growing period (April–October) of 2018, 2019, and the last 60 years.

	1957–2017		2018		2019	
	T (°C)	Rainfall (mm)	T (°C)	Rainfall (mm)	T (°C)	Rainfall (mm)
April	14.1	34.1	17.1	3.0	14.0	31.5
May	19.5	35.0	21.7	69.0	20.0	29.0
June	24.5	20.4	25.3	139.0	26.6	106
July	26.8	19.2	27.7	42.0	27.6	34.3
August	26.1	15.9	26.7	18.0	27.9	106.4
September	22.2	38.5	22.8	40.0	23.3	86.9
October	16.9	60.5	17.2	25.0	18.1	55.6

**Table 2:** Grapevine physiological parameters. Net photosynthesis (P_n_, μmol m^−2^s^−1^), stomatal conductance (g_s_, mmol m^−2^s^−1^), intrinsic water use efficiency (WUE_i_, μmol mol^−1^), and stem water potential (ψ_stem_, MPa) in single leaves of grapevines cv. Roditis subjected to canopy application of kaolin (KL) and zeolite (ZL), compared to untreated vines (Control, C). Measurements were recorded in five fully expanded leaves per vine at veraison and at harvest of 2018 and in two times of the day (morning, 09.00–10.00; and midday, 13.00–14.00). Data are presented as means ± SD of five replicates. Mean values with different lowercase letters (a, b) represent significant differences between treatments in the same period of day (morning/midday) and stage of the season (veraison/maturation). * represents significant differences between the stages of the season within the same period of day (*p* ≤ 0.05, *t*-test).

		Morning			
Vintage/Stage	Treatment	P_n_	g_s_	WUE_i_	ψ_stem_
193 DOY (12 July 2018) Beginning of véraison	C	5.9 ± 2.4 ^b^	122.3 ± 25.5 ^a^	48.2 ± 18.2 ^b^	−0.89 ± 0.0 ^b^
	KL	7.0 ± 1.9 ^b^	126.4 ± 12.1 ^a^	55.0 ± 17.5 ^b^	−0.33 ± 0.1 ^a^
	ZL	11.4 ± 0.2 ^a^	93.6 ± 28.5 ^a^	122.0 ± 36.2 ^a^	−0.43 ± 0.0 ^a^
	Significance	*	ns	*	*
270 DOY (27 September 2018) Harvest	C	3.42 ± 3.04 ^b^	47.7 ± 15.5 ^b^	71.7 ± 5.0 ^b^	−0.21 ± 0.1 ^b^
	KL	5.95 ± 3.94 ^b^	67.8 ± 33.1 ^b^	87.8 ± 2.3 ^b^	−0.18 ± 0.2 ^a^
	ZL	9.63 ± 1.28 ^a^	93.7 ± 11.2 ^a^	102.8 ± 4.2 ^a^	−0.13 ± 0.2 ^a^
	Significance	*	*	*	*
		**Midday**			
193 DOY (12 July 2018) Beginning of véraison	C	3.8 ± 1.5 ^b^	104.4 ± 26.2 ^a^	36.4 ± 12.0 ^b^	−1.98 ± 0.4 ^b^
	KL	4.5 ± 1.3 ^b^	108.4 ± 8.7 ^a^	41.5 ± 14.7 ^b^	−1.20 ± 0.1 ^b^
	ZL	9.8 ± 0.4 ^a^	89.3 ± 10.2 ^a^	109.7 ± 41.4 ^a^	−0.82 ± 0.3 ^a^
	Significance	*	ns	*	*
270 DOY (27 September 2018) Harvest	C	1.94 ± 1.1 ^b^	29.6 ± 9.1 ^b^	65.5 ± 32.5 ^b^	−0.49 ± 0.0 ^a^
	KL	2.95 ± 0.3 ^b^	38.2 ± 5.4 ^b^	77.2 ± 22.1 ^b^	−0.51 ± 0.3 ^a^
	ZL	7.91 ± 0.8 ^a^	72.8 ± 12.2 ^a^	108.5 ± 14.8 ^a^	−0.43 ± 0.2 ^a^
	Significance	*	*	*	ns

**Table 3:** Yield components, cluster characteristics, and fruit composition recorded on cv. Roditis grapevines subjected to canopy application of kaolin (KL) and zeolite (ZL), in comparison with untreated vines (control, C) in 2018 and 2019 at harvest. Values are presented as means ± SD. Mean values with different lowercase letters (a, b, c) represent significant differences between the treatments, in the same year. *, ** and ns represent significance at *p* ≤ 0.05, *p* ≤ 0.01 and no significant differences (*t*-test), respectively. The interaction of year x treatment was not significant. TSS = total soluble solids; TA = titratable acidity.

Vintage	Treatment	Yield per Vine (Kg)	Clusters per Vine (n)	Cluster Weight (g)	Berries per Cluster (Number)	Berry Weight (g)	TSS (°Brix)	TA (g/L)	Must pH	Total Phenols (mg/kg)
2018	C	9.75 ^b^	26.2	372 ^b^	132	2.82 ^b^	18.5	5.16 ^b^	3.43	1751.85 ^a^
	KL	12.51 ^a^	26.4	474 ^a^	135	3.51 ^a^	18.4	5.79 ^a^	3.39	1071.75 ^b^
	ZL	12.12 ^a^	26.4	459 ^a^	132	3.48 ^a^	19.5	5.78 ^a^	3.38	879.53 ^b^
	Significance	*	ns	*	ns	*	ns	*	ns	*
2019	C	11.74 ^c^	24.5	479 ^b^	153	3.13 ^b^	18.6	5.27 ^b^	3.09	1293.79
	KL	12.83 ^b^	25.3	507 ^a^	153	3.31 ^b^	19.1	5.70 ^a^	3.13	1130.96
	ZL	14.28 ^a^	24.5	583 ^a^	163	3.58 ^a^	18.6	5.70 ^a^	3.13	1197.57
	Significance	**	ns	**	ns	*	ns	*	ns	ns

## Data Availability

Not applicable.

## Supplementary Materials

The following supporting information can be downloaded at: https://www.mdpi.com/article/10.3390/plants12071444/s1. Supplementary Figure S1. Typical white residues on Roditis grapevine leaves after kaolin (left) and zeolite applications (right). Photos were taken one hour after the first application.
