# Supplementary material for: Enhancing Yield and Physiological Performance by Foliar Applications of Chemically Inert Mineral Particles in a Rainfed Vineyard under Mediterranean Conditions
Source: Plants (Basel). 2023 Mar 24;12(7):1444. doi: 10.3390/plants12071444 (PMC10096938; doi:10.3390/plants12071444)
Supplement: Supplementary file 1 [file plants-12-01444-s001.zip › plants-2305430-supplementary.pdf]

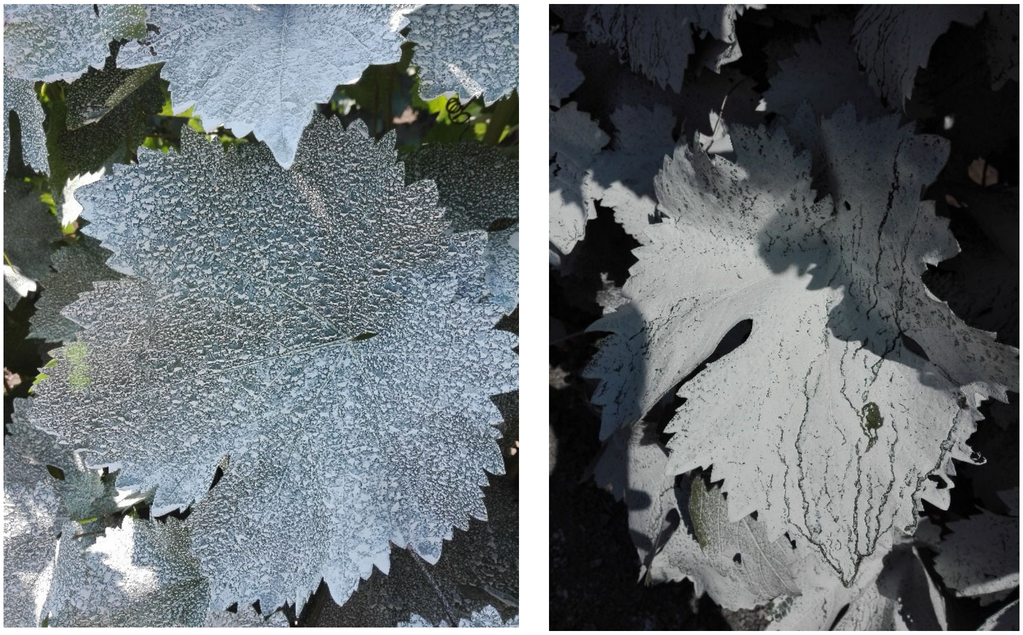

Supplementary Figure S1. Typical white residues on Roditis grapevine leaves after kaolin (left) and zeolite applications (right). Photos were taken one hour after the first application.
